# Long-Term Follow-Up of Transsexual Persons Undergoing Sex Reassignment Surgery: Cohort Study in Sweden

**DOI:** 10.1371/journal.pone.0016885

**Published:** 2011-02-22

**Authors:** Cecilia Dhejne, Paul Lichtenstein, Marcus Boman, Anna L. V. Johansson, Niklas Långström, Mikael Landén

**Affiliations:** 1 Department of Clinical Neuroscience, Division of Psychiatry, Karolinska Institutet, Stockholm, Sweden; 2 Department of Medical Epidemiology and Biostatistics, Karolinska Institutet, Stockholm, Sweden; 3 Centre for Violence Prevention, Karolinska Institutet, Stockholm, Sweden; 4 Institute of Neuroscience and Physiology, The Sahlgrenska Academy at Gothenburg University, Gothenburg, Sweden; The University of Queensland, Australia

## Abstract

**Context:**

The treatment for transsexualism is sex reassignment, including hormonal treatment and surgery aimed at making the person's body as congruent with the opposite sex as possible. There is a dearth of long term, follow-up studies after sex reassignment.

**Objective:**

To estimate mortality, morbidity, and criminal rate after surgical sex reassignment of transsexual persons.

**Design:**

A population-based matched cohort study.

**Setting:**

Sweden, 1973-2003.

**Participants:**

All 324 sex-reassigned persons (191 male-to-females, 133 female-to-males) in Sweden, 1973–2003. Random population controls (10∶1) were matched by birth year and birth sex or reassigned (final) sex, respectively.

**Main Outcome Measures:**

Hazard ratios (HR) with 95% confidence intervals (CI) for mortality and psychiatric morbidity were obtained with Cox regression models, which were adjusted for immigrant status and psychiatric morbidity prior to sex reassignment (adjusted HR [aHR]).

**Results:**

The overall mortality for sex-reassigned persons was higher during follow-up (aHR 2.8; 95% CI 1.8–4.3) than for controls of the same birth sex, particularly death from suicide (aHR 19.1; 95% CI 5.8–62.9). Sex-reassigned persons also had an increased risk for suicide attempts (aHR 4.9; 95% CI 2.9–8.5) and psychiatric inpatient care (aHR 2.8; 95% CI 2.0–3.9). Comparisons with controls matched on reassigned sex yielded similar results. Female-to-males, but not male-to-females, had a higher risk for criminal convictions than their respective birth sex controls.

**Conclusions:**

Persons with transsexualism, after sex reassignment, have considerably higher risks for mortality, suicidal behaviour, and psychiatric morbidity than the general population. Our findings suggest that sex reassignment, although alleviating gender dysphoria, may not suffice as treatment for transsexualism, and should inspire improved psychiatric and somatic care after sex reassignment for this patient group.

## Introduction

Transsexualism (ICD-10),[1] or gender identity disorder (DSM-IV),[2] is a condition in which a person's gender identity - the sense of being a man or a woman - contradicts his or her bodily sex characteristics. The individual experiences gender dysphoria and desires to live and be accepted as a member of the opposite sex.

The treatment for transsexualism includes removal of body hair, vocal training, and cross-sex hormonal treatment aimed at making the person's body as congruent with the opposite sex as possible to alleviate the gender dysphoria. Sex reassignment also involves the surgical removal of body parts to make external sexual characteristics resemble those of the opposite sex, so called sex reassignment/confirmation surgery (SRS). This is a unique intervention not only in psychiatry but in all of medicine. The present form of sex reassignment has been practised for more than half a century and is the internationally recognized treatment to ease gender dysphoria in transsexual persons.[3], [4]

Despite the long history of this treatment, however, outcome data regarding mortality and psychiatric morbidity are scant. With respect to suicide and deaths from other causes after sex reassignment, an early Swedish study followed 24 transsexual persons for an average of six years and reported one suicide.[5] A subsequent Swedish study recorded three suicides after sex reassignment surgery of 175 patients.[6] A recent Swedish follow-up study reported no suicides in 60 transsexual patients, but one death due to complications after the sex reassignment surgery.[7] A Danish study reported death by suicide in 3 out of 29 operated male-to-female transsexual persons followed for an average of six years.[8] By contrast, a Belgian study of 107 transsexual persons followed for 4–6 years found no suicides or deaths from other causes.[9] A large Dutch single-centre study (N = 1,109), focusing on adverse events following hormonal treatment, compared the outcome after cross-sex hormone treatment with national Dutch standardized mortality and morbidity rates and found no increased mortality, with the exception of death from suicide and AIDS in male-to-females 25–39 years of age.[10] The same research group concluded in a recent report that treatment with cross-sex hormones seems acceptably safe, but with the reservation that solid clinical data are missing.[11] A limitation with respect to the Dutch cohort is that the proportion of patients treated with cross-sex hormones who also had surgical sex-reassignment is not accounted for.[10]

Data is inconsistent with respect to psychiatric morbidity post sex reassignment. Although many studies have reported psychiatric and psychological improvement after hormonal and/or surgical treatment,[7], [12], [13], [14], [15], [16] other have reported on regrets,[17] psychiatric morbidity, and suicide attempts after SRS.[9], [18] A recent systematic review and meta-analysis concluded that approximately 80% reported subjective improvement in terms of gender dysphoria, quality of life, and psychological symptoms, but also that there are studies reporting high psychiatric morbidity and suicide rates after sex reassignment.[19] The authors concluded though that the evidence base for sex reassignment “is of very low quality due to the serious methodological limitations of included studies.”

The methodological shortcomings have many reasons. First, the nature of sex reassignment precludes double blind randomized controlled studies of the result. Second, transsexualism is rare [20] and many follow-ups are hampered by small numbers of subjects.[5], [8], [21], [22], [23], [24], [25], [26], [27], [28] Third, many sex reassigned persons decline to participate in follow-up studies, or relocate after surgery, resulting in high drop-out rates and consequent selection bias.[6], [9], [12], [21], [24], [28], [29], [30] Forth, several follow-up studies are hampered by limited follow-up periods.[7], [9], [21], [22], [26], [30] Taken together, these limitations preclude solid and generalisable conclusions. A long-term population-based controlled study is one way to address these methodological shortcomings.

Here, we assessed mortality, psychiatric morbidity, and psychosocial integration expressed in criminal behaviour after sex reassignment in transsexual persons, in a total population cohort study with long-term follow-up information obtained from Swedish registers. The cohort was compared with randomly selected population controls matched for age and gender. We adjusted for premorbid differences regarding psychiatric morbidity and immigrant status. This study design sheds new light on transsexual persons' health after sex reassignment. It does not, however, address whether sex reassignment is an effective treatment or not.

## Methods

### National registers

The study population was identified by the linkage of several Swedish national registers, which contained a total of 13.8 million unique individuals. The Hospital Discharge Register (HDR, held by the National Board of Health and Welfare) contains discharge diagnoses, up to seven contributory diagnoses, external causes of morbidity or mortality, surgical procedure codes, and discharge date. Discharge diagnoses are coded according to the 8^th^ (1969-1986), 9^th^ (1987–1996), and 10^th^ editions (1997-) of the International Classification of Diseases (ICD). The register covers virtually all psychiatric inpatient episodes in Sweden since 1973. Discharges that occurred up to 31 December 2003 were included. Surgical procedure codes could not be used for this study due to the lack of a specific code for sex reassignment surgery. The Total Population Register (TPR, held by Statistics Sweden) is comprised of data about the entire Swedish population. Through linkage with the Total Population Register it was possible to identify birth date and birth gender for all study subjects. The register is updated every year and gender information was available up to 2004/2005. The Medical Birth Register (MBR) was established in 1973 and contains birth data, including gender of the child at birth. National censuses based on mandatory self-report questionnaires completed by all adult citizens in 1960, 1970, 1980, and 1990 provided information on individuals, households, and dwellings, including gender, living area, and highest educational level. Complete migration data, including country of birth for immigrants for 1969–2003, were obtained from the TPR. In addition to educational information from the censuses, we also obtained highest educational level data for 1990 and 2000 from the Register of Education. The Cause of Death Register (CDR, Statistics Sweden) records all deaths in Sweden since 1952 and provided information on date of death and causes of death. Death events occurring up to 31 December 2003 are included in the study. The Crime Register (held by the National Council of Crime Prevention) provided information regarding crime type and date on all criminal convictions in Sweden during the period 1973–2004. Attempted and aggravated forms of all offences were also included. All crimes in Sweden are registered regardless of insanity at the time of perpetration; for example, for individuals who suffered from psychosis at the time of the offence. Moreover, conviction data include individuals who received custodial or non-custodial sentences and cases where the prosecutor decided to caution or fine without court proceedings. Finally, Sweden does not differ considerably from other members of the European Union regarding rates of violent crime and their resolution.[31]

### Study population, identification of sex-reassigned persons (exposure assessment)

The study was designed as a population-based matched cohort study. We used the individual national registration number, assigned to all Swedish residents, including immigrants on arrival, as the primary key through all linkages. The registration number consists of 10 digits; the first six provide information of the birth date, whereas the ninth digit indicates the gender. In Sweden, a person presenting with gender dysphoria is referred to one of six specialised gender teams that evaluate and treat patients principally according to international consensus guidelines: Standards of Care.[3] With a medical certificate, the person applies to the National Board of Health and Welfare to receive permission for sex reassignment surgery and a change of legal sex status. A new national registration number signifying the new gender is assigned after sex reassignment surgery. The National Board of Health and Welfare maintains a link between old and new national registration numbers, making it possible to follow individuals undergoing sex reassignment across registers and over time. Hence, sex reassignment surgery in Sweden requires (i) a transsexualism diagnosis and (ii) permission from the National Board of Health and Welfare.

A person was defined as exposed to sex reassignment surgery if two criteria were met: (i) at least one inpatient diagnosis of gender identity disorder diagnosis without concomitant psychiatric diagnoses in the Hospital Discharge Register, and (ii) at least one discrepancy between gender variables in the Medical Birth Register (from 1973 and onwards) or the National Censuses from 1960, 1970, 1980, or 1990 and the latest gender designation in the Total Population Register. The first criterion was employed to capture the hospitalization for sex reassignment surgery that serves to secure the diagnosis and provide a time point for sex reassignment surgery; the plastic surgeons namely record the reason for sex reassignment surgery, i.e., transsexualism, but not any co-occurring psychiatric morbidity. The second criterion was used to ensure that the person went through all steps in sex-reassignment and also changed sex legally.

The date of sex reassignment (start of follow-up) was defined as the first occurrence of a gender identity disorder diagnosis, without any other concomitant psychiatric disorder, in the Hospital Discharge Register after the patient changed sex status (any discordance in sex designation across the Censuses, Medical Birth, and Total Population registers). If this information was missing, we used instead the closest date in the Hospital Discharge Register on which the patient was diagnosed with gender identity disorder without concomitant psychiatric disorder prior to change in sex status. The reason for prioritizing the use of a gender identity disorder diagnosis *after* changed sex status over *before* was to avoid overestimating person-years at risk of sex-reassigned person.

Using these criteria, a total of 804 patients with gender identity disorder were identified, whereof 324 displayed a shift in the gender variable during the period 1973–2003. The 480 persons that did not shift gender variable comprise persons who either did not apply, or were not approved, for sex reassignment surgery. Moreover, the ICD 9 code 302 is a non specific code for sexual disorders. Hence, this group might also comprise persons that were hospitalized for sexual disorders other than transsexualism. Therefore, they were omitted from further analyses. Of the remaining 324 persons, 288 were identified with the gender identity diagnosis *after* and 36 *before* change of sex status. Out of the 288 persons identified *after* changed sex status, 185 could also be identified *before* change in sex status. The median time lag between the hospitalization *before* and *after* sex change for these 185 persons was 0.96 years (mean 2.2 years, SD 3.3).

Gender identity disorder was coded according to ICD-8: 302.3 (transsexualism) and 302.9 (sexual deviation NOS); ICD-9: 302 (overall code for sexual deviations and disorders, more specific codes were not available in ICD-9); and ICD-10: F64.0 (transsexualism), F64.1 (dual-role transvestism), F64.8 (other gender identity disorder), and F64.9 (gender identity disorder NOS). Other psychiatric disorders were coded as ICD-8: 290-301 and 303-315; ICD-9: 290-301 and 303-319; and ICD-10: F00-F63 as well as F65-F99.

### Identification of population-based controls (unexposed group)

For each exposed person (N = 324), we randomly selected 10 unexposed controls. A person was defined as unexposed if there were no discrepancies in sex designation across the Censuses, Medical Birth, and Total Population registers *and* no gender identity disorder diagnosis according to the Hospital Discharge Register. Control persons were matched by sex and birth year and had to be alive and residing in Sweden at the estimated sex reassignment date of the case person. To study possible gender-specific effects on outcomes of interest, we used two different control groups: one with the same sex as the case individual at birth (birth sex matching) and the other with the sex that the case individual had been reassigned to (final sex matching).

### Outcome measures

We studied mortality, psychiatric morbidity, accidents, and crime following sex reassignment. More specifically, we investigated: (1) all-cause mortality, (2) death by definite/uncertain suicide, (3) death by cardiovascular disease, and (4) death by tumour. Morbidity included (5) any psychiatric disorder (gender identity disorders excluded), (6) alcohol/drug misuse and dependence, (7) definite/uncertain suicide attempt, and (8) accidents. Finally, we addressed court convictions for (9) any criminal offence and (10) any violent offence. Each individual could contribute with several outcomes, but only one event per outcome. Causes of death (Cause of Death Registry from 1952 and onwards) were defined according to ICD as suicide (ICD-8 and ICD-9 codes E950-E959 and E980-E989, ICD-10 codes X60-X84 and Y10-Y34); cardiovascular disease (ICD-8 codes 390-458, ICD-9 codes 390-459, ICD-10 codes I00-I99); neoplasms (ICD-8 and ICD-9 codes 140-239, ICD-10 codes C00-D48), any psychiatric disorder (gender identity disorders excluded); (ICD-8 codes 290-301 and 303-315, ICD-9 codes 290-301 and 303-319, ICD-10 codes F00-F63 and F65-F99); alcohol/drug abuse and dependence (ICD-8 codes 303-304, ICD-9 codes 303-305 (tobacco use disorder excluded), ICD-10 codes F10-F16 and F18-F19 (x5 excluded); and accidents (ICD-8 and ICD-9 codes E800-E929, ICD-10 codes V01-X59).

Any criminal conviction during follow-up was counted; specifically, violent crime was defined as homicide and attempted homicide, aggravated assault and assault, robbery, threatening behaviour, harassment, arson, or any sexual offense.[32]

### Covariates

Severe psychiatric morbidity was defined as inpatient care according to ICD-8 codes 291, 295-301, 303-304, and 307; ICD-9 codes 291-292, 295-298, 300-301, 303-305 (tobacco use disorder excluded), 307.1, 307.5, 308-309, and 311; ICD-10 codes F10-F16, F18-F25, F28-F45, F48, F50, and F60-F62. Immigrant status, defined as individuals born abroad, was obtained from the Total Population Register. All outcome/covariate variables were dichotomized (i.e., affected or unaffected) and without missing values.

### Statistical analyses

Each individual contributed person-time from study entry (for exposed: date of sex reassignment; for unexposed: date of sex reassignment of matched case) until date of outcome event, death, emigration, or end of study period (31 December 2003), whichever came first. The association between exposure (sex reassignment) and outcome (mortality, morbidity, crime) was measured by hazard ratios (HR) with 95% CIs, taking follow-up time into account. HRs were estimated from Cox proportional hazard regression models, stratified on matched sets (1∶10) to account for the matching by sex, age, and calendar time (birth year). We present crude HRs (though adjusted for sex and age through matching) and confounder-adjusted HRs [aHRs] for all outcomes. The two potential confounders, immigrant status (yes/no) and history of severe psychiatric morbidity (yes/no) prior to sex reassignment, were chosen based on previous research[18],[33] and different prevalence across cases and controls (Table 1).

**Table 1:** Baseline characteristics among sex-reassigned subjects in Sweden (N = 324) and population controls matched for birth year and sex.

Characteristic at baseline	Sex-reassigned subjects (N = 324)	Birth-sex matched controls (N = 3,240)	Final-sex matched controls (N = 3,240)
Gender			
Female at birth, male after sex change	133 (41%)	1,330 (41%)	1,330 (41%)
Male at birth, female after sex change	191 (59%)	1,910 (59%)	1,910 (59%)
Average age at study entry [years] (SD, min-max)			
Female at birth, male after sex change	33.3 (8.7, 20–62)	33.3 (8.7, 20–62)	33.3 (8.7, 20–62)
Male at birth, female after sex change	36.3 (10.1, 21–69)	36.3 (10.1, 21–69)	36.3 (10.1, 21–69)
Both genders	35.1 (9.7, 20–69)	35.1 (9.7, 20–69)	35.1 (9.7, 20–69)
Immigrant status			
Female at birth, male after sex change	28 (21%)	118 (9%)	100 (8%)
Male at birth, female after sex change	42 (22%)	176 (9%)	164 (9%)
Both genders	70 (22%)	294 (9%)	264 (8%)
Less than 10 years of schooling prior to entry vs. 10 years or more			
Females at birth, males after sex change	49 (44%); 62 (56%)	414 (37%); 714 (63%)	407 (36%); 713 (64%)
Males at birth, females after sex change	61 (41%); 89 (59%)	665 (40%); 1,011 (60%)	595 (35%); 1,091 (65%)
All individuals with data	110 (42%); 151 (58%)	1,079 (38%); 1,725 (62%)	1,002 (36%); 1,804 (64%)
Psychiatric morbidity* prior to study entry			
Female at birth, male after sex change	22 (17%)	47 (4%)	42 (3%)
Male at birth, female after sex change	36 (19%)	76 (4%)	72 (4%)
Both genders	58 (18%)	123 (4%)	114 (4%)
Rural [vs. urban] living area prior to entry			
Female at birth, male after sex change	13 (10%)	180 (14%)	195 (15%)
Male at birth, female after sex change	20 (10%)	319 (17%)	272 (14%)
Both genders	33 (10%)	499 (15%)	467 (14%)

**Note:**

*Hospitalizations for gender identity disorder were not included.

Gender-separated analyses were performed and a Kaplan-Meier survival plot graphically illustrates the survival of the sex reassigned cohort and matched controls (all-cause mortality) over time. The significance level was set at 0.05 (all tests were two-sided). All outcome/covariate variables were without missing values, since they are generated from register data, which are either present (affected) or missing (unaffected). The data were analysed using SAS version 9.1 (SAS Institute Inc., Cary, NC, USA).

### Ethics

The data linking of national registers required for this study was approved by the IRB at Karolinska Institutet, Stockholm. All data were analyzed anonymously; therefore, informed consent for each individual was neither necessary nor possible.

## Results

We identified 324 transsexual persons (exposed cohort) who underwent sex reassignment surgery and were assigned a new legal sex between 1973 and 2003. These constituted the sex-reassigned (exposed) group. Fifty-nine percent (N = 191) of sex-reassigned persons were male-to-females and 41% (N = 133) female-to-males, yielding a sex ratio of 1.4∶1 (Table 1).

The average follow-up time for all-cause mortality was 11.4 (median 9.1) years. The average follow-up time for the risk of being hospitalized for any psychiatric disorder was 10.4 (median 8.1).

### Characteristics prior to sex reassignment

Table 1 displays demographic characteristics of sex-reassigned and control persons prior to study entry (sex reassignment). There were no substantial differences between female-to-males and male-to-females regarding measured baseline characteristics. Immigrant status was twice as common among transsexual individuals compared to controls, living in an urban area somewhat more common, and higher education about equally prevalent. Transsexual individuals had been hospitalized for psychiatric morbidity other than gender identity disorder prior to sex reassignment about four times more often than controls. To adjust for these baseline discrepancies, hazard ratios adjusted for immigrant status and psychiatric morbidity prior to baseline are presented for all outcomes [aHRs].

### Mortality

**Figure 1:**
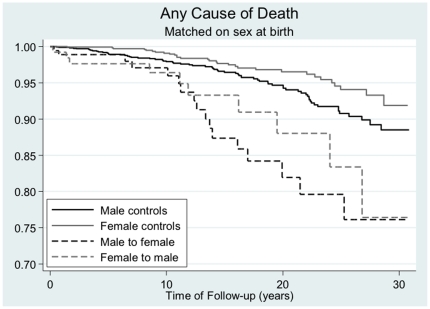
Death from any cause as a function of time after sex reassignment among 324 transsexual persons in Sweden (male-to-female: N = 191, female-to-male: N = 133), and population controls matched on birth year.

Table 2 describes the risks for selected outcomes during follow-up among sex-reassigned persons, compared to same-age controls of the same birth sex. Sex-reassigned transsexual persons of both genders had approximately a three times higher risk of all-cause mortality than controls, also after adjustment for covariates. Table 2 separately lists the outcomes depending on when sex reassignment was performed: during the period 1973-1988 or 1989–2003. Even though the overall mortality was increased across both time periods, it did not reach statistical significance for the period 1989–2003. The Kaplan-Meier curve (Figure 1) suggests that survival of transsexual persons started to diverge from that of matched controls after about 10 years of follow-up. The cause-specific mortality from suicide was much higher in sex-reassigned persons, compared to matched controls. Mortality due to cardiovascular disease was moderately increased among the sex-reassigned, whereas the numerically increased risk for malignancies was borderline statistically significant. The malignancies were lung cancer (N = 3), tongue cancer (N = 1), pharyngeal cancer (N = 1), pancreas cancer (N = 1), liver cancer (N = 1), and unknown origin (N = 1).

**Table 2:** Risk of various outcomes among sex-reassigned subjects in Sweden (N = 324) compared to population controls matched for birth year and birth sex.

	Number of eventscases/controls1973–2003	Outcome incidence rateper 1000 person-years1973–2003(95% CI)		Crudehazard ratio(95% CI)1973–2003	Adjusted*hazard ratio(95% CI)1973–2003	Adjusted*hazard ratio(95% CI)1973–1988	Adjusted*hazard ratio(95% CI)1989–2003
		Cases	Controls				
Any death	27/99	7.3 (5.0–10.6)	2.5 (2.0–3.0)	2.9 (1.9–4.5)	2.8 (1.8–4.3)	3.1 (1.9–5.0)	1.9 (0.7–5.0)
Death by suicide	10/5	2.7 (1.5–5.0)	0.1 (0.1–0.3)	19.1 (6.5–55.9)	19.1 (5.8–62.9)	N/A	N/A
Death by cardiovasculardisease	9/42	2.4 (1.3–4.7)	1.1 (0.8–1.4)	2.6 (1.2–5.4)	2.5 (1.2–5.3)	N/A	N/A
Death by neoplasm	8/38	2.2 (1.1–4.3)	1.0 (0.7–1.3)	2.1 (1.0–4.6)	2.1 (1.0–4.6)	N/A	N/A
Any psychiatrichospitalisation‡	64/173	19.0 (14.8–24.2)	4.2 (3.6–4.9)	4.2 (3.1–5.6)	2.8 (2.0–3.9)	3.0 (1.9–4.6)	2.5 (1.4–4.2)
Substance misuse	22/78	5.9 (3.9–8.9)	1.8 (1.5–2.3)	3.0 (1.9–4.9)	1.7 (1.0–3.1)	N/A	N/A
Suicide attempt	29/44	7.9 (5.5–11.4)	1.0 (0.8–1.4)	7.6 (4.7–12.4)	4.9 (2.9–8.5)	7.9 (4.1–15.3)	2.0 (0.7–5.3)
Any accident	32/233	9.0 (6.3–12.7)	5.7 (5.0–6.5)	1.6 (1.1–2.3)	1.4 (1.0–2.1)	1.6 (1.0–2.5)	1.1 (0.5–2.2)
Any crime	60/350	18.5 (14.3–23.8)	9.0 (8.1–10.0)	1.9 (1.4–2.5)	1.3 (1.0–1.8)	1.6 (1.1–2.4)	0.9 (0.6–1.5)
Violent crime	14/61	3.6 (2.1–6.1)	1.4 (1.1–1.8)	2.7 (1.5–4.9)	1.5 (0.8–3.0)	N/A	N/A

**Notes:**

*Adjusted for psychiatric morbidity prior to baseline and immigrant status.

‡ Hospitalisations for gender identity disorder were excluded.

N/A Not applicable due to sparse data.

### Psychiatric morbidity, substance misuse, and accidents

Sex-reassigned persons had a higher risk of inpatient care for a psychiatric disorder other than gender identity disorder than controls matched on birth year and birth sex (Table 2). This held after adjustment for prior psychiatric morbidity, and was true regardless of whether sex reassignment occurred before or after 1989. In line with the increased mortality from suicide, sex-reassigned individuals were also at a higher risk for suicide attempts, though this was not statistically significant for the time period 1989–2003. The risks of being hospitalised for substance misuse or accidents were not significantly increased after adjusting for covariates (Table 2).

### Crime rate

Transsexual individuals were at increased risk of being convicted for any crime or violent crime after sex reassignment (Table 2); this was, however, only significant in the group who underwent sex reassignment before 1989.

### Gender differences

Comparisons of female-to-males and male-to-females, although hampered by low statistical power and associated wide confidence intervals, suggested mostly similar risks for adverse outcomes (Tables S1 and S2). However, violence against self (suicidal behaviour) and others ([violent] crime) constituted important exceptions. First, male-to-females had significantly increased risks for suicide attempts compared to both female (aHR 9.3; 95% CI 4.4–19.9) and male (aHR 10.4; 95% CI 4.9–22.1) controls. By contrast, female-to-males had significantly increased risk of suicide attempts only compared to male controls (aHR 6.8; 95% CI 2.1–21.6) but not compared to female controls (aHR 1.9; 95% CI 0.7–4.8). This suggests that male-to-females are at higher risk for suicide attempts after sex reassignment, whereas female-to-males maintain a female pattern of suicide attempts after sex reassignment (Tables S1 and S2).

Second, regarding any crime, male-to-females had a significantly increased risk for crime compared to female controls (aHR 6.6; 95% CI 4.1–10.8) but not compared to males (aHR 0.8; 95% CI 0.5–1.2). This indicates that they retained a male pattern regarding criminality. The same was true regarding violent crime. By contrast, female-to-males had higher crime rates than female controls (aHR 4.1; 95% CI 2.5–6.9) but did not differ from male controls. This indicates a shift to a male pattern regarding criminality and that sex reassignment is coupled to increased crime rate in female-to-males. The same was true regarding violent crime.

## Discussion

### Principal findings and comparison with previous research

We report on the first nationwide population-based, long-term follow-up of sex-reassigned transsexual persons. We compared our cohort with randomly selected population controls matched for age and gender. The most striking result was the high mortality rate in both male-to-females and female-to males, compared to the general population. This contrasts with previous reports (with one exception[8]) that did not find an increased mortality rate after sex reassignment, or only noted an increased risk in certain subgroups.[7], [9], [10], [11] Previous clinical studies might have been biased since people who regard their sex reassignment as a failure are more likely to be lost to follow-up. Likewise, it is cumbersome to track deceased persons in clinical follow-up studies. Hence, population-based register studies like the present are needed to improve representativity.[19], [34]

The poorer outcome in the present study might also be explained by longer follow-up period (median >10 years) compared to previous studies. In support of this notion, the survival curve (Figure 1) suggests increased mortality from ten years after sex reassignment and onwards. In accordance, the overall mortality rate was only significantly increased for the group operated before 1989. However, the latter might also be explained by improved health care for transsexual persons during 1990s, along with altered societal attitudes towards persons with different gender expressions.[35]

Mortality due to cardiovascular disease was significantly increased among sex reassigned individuals, albeit these results should be interpreted with caution due to the low number of events. This contrasts, however, a Dutch follow-up study that reported no increased risk for cardiovascular events.[10], [11] A recent meta-analysis concluded, however, that data on cardiovascular outcome after cross-sex steroid use are sparse, inconclusive, and of very low quality.[34]

With respect to neoplasms, prolonged hormonal treatment might increase the risk for malignancies,[36] but no previous study has tested this possibility. Our data suggested that the cause-specific risk of death from neoplasms was increased about twice (borderline statistical significance). These malignancies (see Results), however, are unlikely to be related to cross-hormonal treatment.

There might be other explanations to increased cardiovascular death and malignancies. Smoking was in one study reported in almost 50% by the male-to females and almost 20% by female-to-males.[9] It is also possible that transsexual persons avoid the health care system due to a presumed risk of being discriminated.

Mortality from suicide was strikingly high among sex-reassigned persons, also after adjustment for prior psychiatric morbidity. In line with this, sex-reassigned persons were at increased risk for suicide attempts. Previous reports [6], [8], [10], [11] suggest that transsexualism is a strong risk factor for suicide, also after sex reassignment, and our long-term findings support the need for continued psychiatric follow-up for persons at risk to prevent this.

Inpatient care for psychiatric disorders was significantly more common among sex-reassigned persons than among matched controls, both before and after sex reassignment. It is generally accepted that transsexuals have more psychiatric ill-health than the general population prior to the sex reassignment.[18], [21], [22], [33] It should therefore come as no surprise that studies have found high rates of depression,[9] and low quality of life[16], [25] also after sex reassignment. Notably, however, in this study the increased risk for psychiatric hospitalisation persisted even after adjusting for psychiatric hospitalisation prior to sex reassignment. This suggests that even though sex reassignment alleviates gender dysphoria, there is a need to identify and treat co-occurring psychiatric morbidity in transsexual persons not only before but also after sex reassignment.

Criminal activity, particularly violent crime, is much more common among men than women in the general population. A previous study of all applications for sex reassignment in Sweden up to 1992 found that 9.7% of male-to-female and 6.1% of female-to-male applicants had been prosecuted for a crime.[33] Crime after sex reassignment, however, has not previously been studied. In this study, male-to-female individuals had a higher risk for criminal convictions compared to female controls but not compared to male controls. This suggests that the sex reassignment procedure neither increased nor decreased the risk for criminal offending in male-to-females. By contrast, female-to-males were at a higher risk for criminal convictions compared to female controls and did not differ from male controls, which suggests increased crime proneness in female-to-males after sex reassignment.

### Strengths and limitations of the study

Strengths of this study include nationwide representativity over more than 30 years, extensive follow-up time, and minimal loss to follow-up. Many previous studies suffer from low outcome ascertainment,[6], [9], [21], [29] whereas this study has captured almost the entire population of sex-reassigned transsexual individuals in Sweden from 1973–2003. Moreover, previous outcome studies have mixed pre-operative and post-operative transsexual persons,[22], [37] while we included only post-operative transsexual persons that also legally changed sex. Finally, whereas previous studies either lack a control group or use standardised mortality rates or standardised incidence rates as comparisons,[9], [10], [11] we selected random population controls matched by birth year, and either birth or final sex.

Given the nature of sex reassignment, a double blind randomized controlled study of the result after sex reassignment is not feasible. We therefore have to rely on other study designs. For the purpose of evaluating whether sex reassignment is an effective treatment for gender dysphoria, it is reasonable to compare reported gender dysphoria pre and post treatment. Such studies have been conducted either prospectively[7], [12] or retrospectively,[5], [6], [9], [22], [25], [26], [29], [38] and suggest that sex reassignment of transsexual persons improves quality of life and gender dysphoria. The limitation is of course that the treatment has not been assigned randomly and has not been carried out blindly.

For the purpose of evaluating the safety of sex reassignment in terms of morbidity and mortality, however, it is reasonable to compare sex reassigned persons with matched population controls. The caveat with this design is that transsexual persons before sex reassignment might differ from healthy controls (although this bias can be statistically corrected for by adjusting for baseline differences). It is therefore important to note that the current study is only informative with respect to transsexuals persons health after sex reassignment; no inferences can be drawn as to the effectiveness of sex reassignment as a treatment for transsexualism. In other words, the results should not be interpreted such as sex reassignment *per se* increases morbidity and mortality. Things might have been even worse without sex reassignment. As an analogy, similar studies have found increased somatic morbidity, suicide rate, and overall mortality for patients treated for bipolar disorder and schizophrenia.[39], [40] This is important information, but it does not follow that mood stabilizing treatment or antipsychotic treatment is the culprit.

Other facets to consider are first that this study reflects the outcome of psychiatric and somatic treatment for transsexualism provided in Sweden during the 1970s and 1980s. Since then, treatment has evolved with improved sex reassignment surgery, refined hormonal treatment,[11], [41] and more attention to psychosocial care that might have improved the outcome. Second, transsexualism is a rare condition and Sweden is a small country (9.2 million inhabitants in 2008). Hence, despite being based on a comparatively large national cohort and long-term follow-up, the statistical power was limited. Third, regarding psychiatric morbidity after sex reassignment, we assessed inpatient psychiatric care. Since most psychiatric care is provided in outpatient settings (for which no reliable data were available), underestimation of the *absolute* prevalences was inevitable. However, there is no reason to believe that this would change the *relative risks* for psychiatric morbidity unless sex-reassigned transsexual individuals were more likely than matched controls to be admitted to hospital for any given psychiatric condition.

Finally, to estimate start of follow-up, we prioritized using the date of a gender identity disorder diagnosis *after* changed sex status over *before* changed sex status, in order to avoid overestimating person-years at risk after sex-reassignment. This means that adverse outcomes might have been underestimated. However, given that the median time lag between the hospitalization before and after change of sex status was less than a year (see Methods), this maneuver is unlikely to have influenced the results significantly. Moreover, all deaths will be recorded regardless of this exercise and mortality hence correctly estimated.

### Conclusion

This study found substantially higher rates of overall mortality, death from cardiovascular disease and suicide, suicide attempts, and psychiatric hospitalisations in sex-reassigned transsexual individuals compared to a healthy control population. This highlights that post surgical transsexuals are a risk group that need long-term psychiatric and somatic follow-up. Even though surgery and hormonal therapy alleviates gender dysphoria, it is apparently not sufficient to remedy the high rates of morbidity and mortality found among transsexual persons. Improved care for the transsexual group after the sex reassignment should therefore be considered.

## Supporting Information

Table S1 **Risk of various outcomes in sex-reassigned persons in Sweden compared to population controls matched for birth year and** ***birth sex*** **.**
(DOCX)

Table S2 **Risk of various outcomes in sex-reassigned persons in Sweden compared to controls matched for birth year and** ***final sex*** **.**
(DOCX)

## References

[1] World Health Organization (1993). The ICD-10 Classification of Mental and Behavioural Disorders.. Diagnostic criteria for research.

[2] American Psychiatric Association (1994). Diagnostic and Statistical Manual of Mental Disorders..

[3] Meyer W, Bockting W, Cohen-Kettenis P, Coleman E, DiCeglie D (2002). The Harry Benjamin International Gender Dysphoria Association's Standards of Care for Gender Identity Disorders, Sixth Version.. Journal of Psychology & Human Sexuality.

[4] Cohen-Kettenis PT, Gooren LJG (1999). Transsexualism: A review of etiology, diagnosis and treatment.. J Psychosom Res.

[5] Wålinder J, Thuwe I (1975). A social-psychiatric follow-up study of 24 sex-reassigned transsexuals..

[6] Eldh J, Berg A, Gustafsson M (1997). Long-term follow up after sex reassignment surgery.. Scand J Plast Reconstr Surg Hand Surg.

[7] Johansson A, Sundbom E, Höjerback T, Bodlund O (2010). A five-year follow-up study of Swedish adults with gender identity disorder.. Arch Sex Behav.

[8] Sørensen T, Hertoft P (1982). Male and female transsexualism: the Danish experience with 37 patients.. ArchSex Behav.

[9] De Cuypere G, T'Sjoen G, Beerten R, Selvaggi G, De Sutter P (2005). Sexual and physical health after sex reassignment surgery.. Arch Sex Behav.

[10] van Kesteren PJ, Asscheman H, Megens JA, Gooren LJ (1997). Mortality and morbidity in transsexual subjects treated with cross-sex hormones.. Clin Endocrinol Oxf.

[11] Gooren LJ, Giltay EJ, Bunck MC (2008). Long-term treatment of transsexuals with cross-sex hormones: extensive personal experience.. J Clin Endocrinol Metab.

[12] Smith YL, van Goozen SH, Cohen-Kettenis PT (2001). Adolescents with gender identity disorder who were accepted or rejected for sex reassignment surgery: a prospective follow-up study.. J Am Acad Child Adolesc Psychiatry.

[13] Smith YL, Van Goozen SH, Kuiper AJ, Cohen-Kettenis PT (2005). Sex reassignment: outcomes and predictors of treatment for adolescent and adult transsexuals.. Psychol Med.

[14] Leavitt F, Berger JC, Hoeppner JA, Northrop G (1980). Presurgical adjustment in male transsexuals with and without hormonal treatment.. J Nerv Ment Dis.

[15] Cohen Kettenis PT, van Goozen SH (1997). Sex reassignment of adolescent transsexuals: a follow-up study.. J Am Acad Child Adolesc Psychiatry.

[16] Newfield E, Hart S, Dibble S, Kohler L (2006). Female-to-male transgender quality of life.. Qual Life Res.

[17] Landén M, Wålinder J, Hambert G, Lundström B (1998). Factors predictive of regret in sex reassignment.. Acta Psychiatrica Scandinavica.

[18] Hepp U, Kraemer B, Schnyder U, Miller N, Delsignore A (2005). Psychiatric comorbidity in gender identity disorder.. J Psychosom Res.

[19] Murad MH, Elamin MB, Garcia MZ, Mullan RJ, Murad A (2010). Hormonal therapy and sex reassignment: a systematic review and meta-analysis of quality of life and psychosocial outcomes.. Clin Endocrinol (Oxf).

[20] Landén M, Wålinder J, Lundström B (1996). Incidence and sex ratio of transsexualism in Sweden.. Acta Psychiatrica Scandinavica.

[21] Lobato MI, Koff WJ, Manenti C, da Fonseca Seger D, Salvador J (2006). Follow-up of sex reassignment surgery in transsexuals: a Brazilian cohort.. Arch Sex Behav.

[22] Bodlund O, Kullgren G (1996). Transsexualism-General outcome and prognostic factors. A five year follow-up study of 19 transsexuals in the process of changing sex.. Arch Sex Behav.

[23] Lindemalm G, Körlin D, Uddenberg N (1986). Long-term follow-up of "sex change" in 13 male-to-female transsexuals.. Arch Sex Behav.

[24] Rauchfleisch U, Barth D, Battegay R (1998). [Results of long-term follow-up of transsexual patients].. Nervenarzt.

[25] Kuhn A, Bodmer C, Stadlmayr W, Kuhn P, Mueller MD (2009). Quality of life 15 years after sex reassignment surgery for transsexualism.. Fertil Steril.

[26] Zimmermann A, Zimmer R, Kovacs L, Einodshofer S, Herschbach P (2006). [Transsexuals' life satisfaction after gender transformation operations].. Chirurg.

[27] Rehman J, Lazer S, Benet AE, Schaefer LC, Melman A (1999). The reported sex and surgery satisfactions of 28 postoperative male-to- female transsexual patients.. Arch Sex Behav.

[28] Hepp U, Klaghofer R, Burkhard-Kubler R, Buddeberg C (2002). [Treatment follow-up of transsexual patients. A catamnestic study].. Nervenarzt.

[29] Lawrence AA (2003). Factors associated with satisfaction or regret following male-to-female sex reassignment surgery.. Arch Sex Behav.

[30] Kaube H, Biemer E (1991). [Results of sex change operations in 30 transsexual patients: psychosocial and sexual adaptation–surgical complications].. Handchir Mikrochir Plast Chir.

[31] Dolmén L (2001). Brottsligheten i olika länder (Criminality in different countries)..

[32] Fazel S, Grann M (2006). The population impact of severe mental illness on violent crime.. Am J Psychiatry.

[33] Landén M, Wålinder J, Lundström B (1998). Clinical characteristics of a total cohort of female and male applicants for sex reassignment: a descriptive study.. Acta Psychiatrica Scandinavica.

[34] Elamin MB, Garcia MZ, Murad MH, Erwin PJ, Montori VM (2010). Effect of sex steroid use on cardiovascular risk in transsexual individuals: a systematic review and meta-analyses.. Clin Endocrinol (Oxf).

[35] Landén M, Innala S (2000). Attitudes toward transsexualism in a Swedish national survey.. Archives of Sexual Behavior.

[36] Mueller A, Gooren L (2008). Hormone-related tumors in transsexuals receiving treatment with cross-sex hormones.. Eur J Endocrinol.

[37] Vujovic S, Popovic S, Sbutega-Milosevic G, Djordjevic M, Gooren L (2009). Transsexualism in Serbia: a twenty-year follow-up study.. J Sex Med.

[38] Rehman J, Lazer S, Benet AE, Schaefer LC, Melman A (1999). The reported sex and surgery satisfactions of 28 postoperative male-to-female transsexual patients.. Arch Sex Behav.

[39] Ösby U, Brandt L, Correia N, Ekbom A, Sparén P (2001). Excess mortality in bipolar and unipolar disorder in Sweden.. Arch Gen Psychiatry.

[40] Tidemalm D, Langstrom N, Lichtenstein P, Runeson B (2008). Risk of suicide after suicide attempt according to coexisting psychiatric disorder: Swedish cohort study with long term follow-up.. Bmj.

[41] Toorians AW, Thomassen MC, Zweegman S, Magdeleyns EJ, Tans G (2003). Venous thrombosis and changes of hemostatic variables during cross-sex hormone treatment in transsexual people.. J Clin Endocrinol Metab.

