# Supplementary material for: Long-Term Follow-Up of Transsexual Persons Undergoing Sex Reassignment Surgery: Cohort Study in Sweden
Source: PLoS One. 2011 Feb 22;6(2):e16885. doi: 10.1371/journal.pone.0016885 (PMC3043071; doi:10.1371/journal.pone.0016885)
Supplement: Table S1 — Risk of various outcomes in sex-reassigned persons in Sweden compared to population controls matched for birth year and birth sex . (DOCX) [file pone.0016885.s001.docx]

| **Table S1.** Risk of various outcomes in sex-reassigned subjects in Sweden compared to population controls matched for birth year and *birth sex*. | | | | | | | | |
| --- | --- | --- | --- | --- | --- | --- | --- | --- |
| **Outcome** | | **No. of events**  **(male-to-female/female-to-male)** | **Crude hazard ratio (95% CI)** | | | **Adjusted* hazard ratio (95% CI)** | | |
|  |  |  | **All sex-reassignment persons**  **(N=324)** | **Male-to-female only**  **(N=191)** | **Female-to-male only**  **(N=133)** | **All sex- reassignment persons**  **(N=324)** | **Male-to-female only**  **(N=191)** | **Female-to-male only**  **(N=133)** |
| Any death | | 27 (17/10) | 2.9  (1.9-4.5) | 2.6  (1.5-4.5) | 3.7  (1.8-7.7) | 2.8  (1.8-4.3) | 2.4  (1.4-4.1) | 3.8  (1.8-7.9) |
|  | Death by suicide | 10 (6/4) | 19.1  (6.5-55.9) | 13.9  (3.9-49.6) | 40.0  (4.5-357.9) | N/A | N/A | N/A |
|  | Death by cardiovascular disease | 9 (6/3) | 2.6  (1.2-5.4) | 2.3  (0.9-5.7) | 3.2  (0.9-11.9) | N/A | N/A | N/A |
|  | Death by neoplasm | 8 (4/4) | 2.1  (1.0-4.6) | 1.7  (0.6-4.9) | 2.8  (0.9-8.5) | N/A | N/A | N/A |
| Any psychiatric hospitalisation‡ | | 64 (43/21) | 4.2  (3.1-5.6) | 4.7  (3.2-6.7) | 3.4  (2.1-5.6) | 2.8  (2.0-3.9) | 3.2  (2.1-4.9) | 2.2  (1.3-4.0) |
|  | Substance misuse | 22 (14/8) | 3.0  (1.9-4.9) | 2.8  (1.6-5.1) | 3.5  (1.6-7.8) | 1.7  (1.0-3.1) | 1.5  (0.7-3.1) | 2.3  (0.9-5.8) |
| Suicide attempt | | 29 (22/7) | 7.6  (4.7-12.4) | 15.4  (7.9-30.2) | 2.9  (1.3-6.8) | 4.9  (2.9-8.5) | 10.4  (4.9-22.1) | 1.9  (0.7-4.8) |
| Any accident | | 32 (19/13) | 1.6  (1.1-2.3) | 1.4  (0.9-2.2) | 1.9  (1.0-3.4) | 1.4  (1.0-2.1) | 1.2  (0.7-2.0) | 1.8  (1.0-3.3) |
| Any crime | | 60 (33/27) | 1.9  (1.4-2.5) | 1.2  (0.8-1.7) | 5.6  (3.5-9.1) | 1.3  (1.0-1.8) | 0.8  (0.5-1.2) | 4.1  (2.5-6.9) |
|  | Violent crime | 14 (8/6) | 2.7  (1.5-4.9) | 1.8  (0.8-3.7) | 9.9  (3.2-30.7) | 1.5  (0.8-3.0) | 0.8  (0.3-2.1) | 7.2  (2.1-24.4) |
| **Notes:** N/A Not applicable due to sparse data. *Adjusted for immigrant status and psychiatric morbidity up to baseline. ‡ Hospitalisations for gender identity disorder were excluded. | | | | | | | | |
