# Supplementary material for: Long-Term Follow-Up of Transsexual Persons Undergoing Sex Reassignment Surgery: Cohort Study in Sweden
Source: PLoS One. 2011 Feb 22;6(2):e16885. doi: 10.1371/journal.pone.0016885 (PMC3043071; doi:10.1371/journal.pone.0016885)
Supplement: Table S2 — Risk of various outcomes in sex-reassigned persons in Sweden compared to controls matched for birth year and final sex . (DOCX) [file pone.0016885.s002.docx]

| **Table S2.** Risk of various outcomes in sex-reassigned individuals in Sweden compared to controls matched for birth year and *final sex*. | | | | | | | | |
| --- | --- | --- | --- | --- | --- | --- | --- | --- |
| **Outcome** | | **No. of events**  **(male-to-female/male-to-male)** | **Crude hazard ratio (95% CI)** | | | **Adjusted* hazard ratio (95% CI)** | | |
|  |  |  | **All sex-reassigned subjects**  **(N=324)** | **Male-to-female only**  **(N=191)** | **Female-to-male only**  **(N=133)** | **All sex-reassigned subjects**  **(N=324)** | **Male-to-female only**  **(N=191)** | **Female-to-male only**  **(N=133)** |
| Any death | | 27 (17/10) | 3.2  (2.1-5.0) | 4.5  (2.5-8.0) | 2.2  (1.1-4.3) | 2.9  (1.8-4.6) | 4.2  (2.3-7.7) | 2.2  (1.1-4.6) |
|  | Death from suicide | 10 (6/4) | 12.1  (4.8-30.8) | 60.0  (7.2-498.4) | 5.4  (1.6-18.6) | N/A | N/A | N/A |
|  | Death from cardiovascular disease | 9 (6/3) | 3.8  (1.7-8.2) | 6.8  (2.3-19.6) | 2.0  (0.6-6.9) | N/A | N/A | N/A |
|  | Death from neoplasm | 8 (4/4) | 2.6  (1.2-5.6) | 2.1  (0.7-6.3) | 3.2  (1.0-10.1) | N/A | N/A | N/A |
| Any psychiatric hospitalisation‡ | | 64 (43/21) | 4.3  (3.2-5.8) | 5.4  (3.7-7.8) | 3.1  (1.9-5.1) | 2.9  (2.1-4.2) | 3.7  (2.4-5.6) | 2.1  (1.1-3.8) |
|  | Substance misuse | 22 (14/8) | 4.3  (2.6-7.1) | 6.3  (3.2-12.3) | 2.7  (1.2-6.0) | 2.3  (1.2-4.5) | 2.8  (1.2-6.4) | 2.1  (0.7-6.0) |
| Suicide attempt | | 29 (22/7) | 8.4  (5.1-13.8) | 10.9  (5.9-20.0) | 4.9  (2.0-12.1) | 7.7  (4.2-14.3) | 9.3  (4.4-19.9) | 6.8  (2.1-21.6) |
| Any accident | | 32 (19/13) | 1.4  (1.0-2.1) | 1.7  (1.1-2.8) | 1.2  (0.6-2.1) | 1.2  (0.8-1.8) | 1.5  (0.9-2.6) | 0.9  (0.5-1.8) |
| Any crime | | 60 (33/27) | 2.1  (1.6-2.8) | 7.4  (4.7-11.7) | 1.1  (0.8-1.7) | 1.6  (1.2-2.2) | 6.6  (4.1-10.8) | 0.7  (0.5-1.1) |
|  | Violent crime | 14 (8/6) | 2.3  (1.3-4.1) | 20.0  (6.0-66.4) | 1.1  (0.5-2.4) | 1.7  (0.9-3.1) | 18.1  (5.4-61.2) | 0.6  (0.2-1.6) |
| **Notes:** N/A Not applicable due to sparse data. *Adjusted for immigrant status and psychiatric morbidity up to baseline. ‡ Hospitalisations for gender identity disorder were excluded. | | | | | | | | |
